# Methamphetamine and neuroHIV suppress astrocytic potassium channel function in the medial prefrontal cortex via different mechanisms

**DOI:** 10.3389/fphar.2025.1691165

**Published:** 2025-11-24

**Authors:** Lihua Chen, Stefanie L. Cassoday, Joao I. Mamede, Lena Al-Harthi, Xiu-Ti Hu

**Affiliations:** Department of Microbial Pathogens and Immunity, RUSH University Medical Center, Chicago, IL, United States

**Keywords:** astrocyte, methamphetamine (METH), neuroHIV, medial prefrontal cortex (mPFC), K^+^ channel, electrophysiology, trace amine-associated receptor 1 (TAAR1)

## Abstract

**Introduction:**

Methamphetamine (Meth) is a highly addictive psychostimulant that disrupts neuronal function in the medial prefrontal cortex (mPFC), inducing Meth use disorders (MUD). MUD is often complicated by HIV-associated neurocognitive disorders (HAND, a.k.a. neuroAIDS/neuroHIV), and *vice versa*. MUD and neuroHIV also disrupt astrocytes, altering their role in supporting normal neuron function. The mechanism(s) underlying Meth and neuroHIV’s impact on astrocytes and astrocyte-neuron interplay remains unknown.

**Methods:**

To define that, we assessed the activity of cortical astrocyte K^+^ channels that regulate extracellular K^+^ homeostasis ([K^+^]_e_), and substantially neuronal excitability in the brain. HIV-1 transgenic (Tg) rats, a rodent model of neuroHIV with combined antiretroviral therapy (cART) that have no active HIV-1 replication but expression of viral proteins, were given daily repeated Meth administrations. Saline-pretreated non-Tg rats served as control. We then conducted electrophysiological assessment in mPFC astrocytes after acute Meth (20, 100 μM in bath) or daily repeated Meth administrations (5 mg/kg/day s.c. for 5 days) followed by a 3-day withdrawal.

**Results:**

We found that both Meth and neuroHIV suppressed the activity of astrocytic K^+^ channels, regardless of subtypes. The maximum reduction occurred in response to combined Meth/neuroHIV, showing exacerbated astrocyte dysfunction. Blocking the trace amine-associated receptor 1 (TAAR1)/G protein-coupled signaling pathway abolished Meth-induced, but not neuroHIV-induced, suppression of K_2P_, K_v_, and K_ir_ channel activity.

**Discussion:**

Collectively, these findings demonstrate that Meth and neuroHIV inhibit astrocyte function, which could exacerbate mPFC neuronal dysfunction in MUD and/or neuroHIV. They also suggest that Meth- and neuroHIV-induced astrocytic K^+^ channel function was mediated by TAAR1-and/or chemokine receptor-coupled signaling pathways.

## 1 Introduction

Meth is a potent and highly addictive psychostimulant that is widely abused in the world (Cadet and Krasnova, 2009; Glasner-Edwards and Mooney, 2014). To date, there is no medication approved by the Federal Drug and Food administration (FDA) of the United States for treatment of Meth Use disorders (MUD). Chronic exposure to Meth *in vivo* profoundly alters the functional activity of neurons in certain key brain regions, including the medial prefrontal cortex (mPFC), which plays a critical role in addiction and neurocognition (Rusyniak, 2013; Volkow et al., 2001). Thus, mPFC dysfunction has been considered as a major contributor to the neuropathophysiology of MUD (Koob and Volkow, 2016; Goldstein and Volkow, 2011). Moreover, Meth abuse is also prevalent among people diagnosed with HIV-associated neurocognitive disorders (HAND), a.k.a. neuroAIDS, or neuroHIV in animal study. The comorbidity of MUD and neuroHIV could worsen mPFC dysfunction induced by either one alone, leading to more severe cognitive deficits (Ellis et al., 2003; Durvasula and Miller, 2014; Potula and Persidsky, 2008).

Astrocytes is a class of glial cells in the central nerve system (CNS), which are integral to regulating immune responses and maintaining brain homeostasis (Barres, 2008; Sofroniew and Vinters, 2010). Besides their critical role in the immune system, astrocytes are also vital for supporting the normal activity of neurons, particularly by maintaining and regulating extracellular glutamate ([glut]_o_) and potassium ([K^+^]_o_) levels (Cheung et al., 2015; Kadala et al., 2015; Beardsley and Hauser, 2014; Olsen and Sontheimer, 2008; Verkhratsky and Nedergaard, 2018). Such functions of astrocytes are essential for preserving the membrane potential and excitability of nearby neurons in the CNS (Seifert et al., 2016; Seifert et al., 2009; Bellot-Saez et al., 2017; Cheung et al., 2015).

Chronic Meth exposure and neuroHIV disrupts these functions of cortical astrocytes, impairing normal activity of surrounding cortical neurons and exacerbating the dysfunction caused by either condition alone. Acute Meth exposure significantly reduces functional activity of K^+^ channels in cultured human fetal astrocytes mediated by trans-amine associated receptor 1 (TAAR1) and G protein-mediated signaling pathway (Dave et al., 2019). Additionally, chronic exposure to Meth (or other drugs of abuse) and persisting neuroHIV conditions impair cortical astrocytic and neuronal functions, thereby disrupting their interactions and inducing more severe deficits than that caused by each one alone (Potula and Persidsky, 2008; Pendyala et al., 2012; Cisneros and Ghorpade, 2012; Hauser and Knapp, 2014).

Despite these findings, significant gaps remain in understanding whether and how chronic Meth and neuroHIV disturb the dynamic activity of astrocytes and its regulation of [glut]_o_ and [K^+^]_o_ homeostasis in the mPFC. Such Meth and neuroHIV-induced disruption could interrupt synaptic and intrinsic excitability of cortical pyramidal neurons in the brain, particularly by altering astrocytic K^+^ channel activity and glutamate reuptake. Addressing these knowledge gaps will advance our understanding of the mechanism(s) underlying astrocytic and neuronal dysfunctions in the context of MUD, neuroHIV, and comorbidity of both.

To investigate the astrocyte dysfunction in regulating [K^+^] homeostasis (a.k.a. K^+^ buffering) following daily repeated Meth administration, with or without neuroHIV, we assessed functional activity of various K^+^ channel families in live mPFC astrocytes using HIV-1 transgenic (Tg) rats, a rodent model of neuroHIV that have received combined antiretroviral therapy (cART). These animals express seven of nine HIV-1 proteins in various HIV reservoirs, but without active HIV replication, in the central and peripheral nervous systems (Peng et al., 2010; Reid et al., 2001). Although neurons are not infected by HIV, these neurotoxic viral proteins are known to disrupt mPFC neuronal activity (Khodr et al., 2016; Khodr et al., 2022; Chen et al., 2019), inducing dysfunction (i.e., hyperactivity and overactivation) and injury of cortical pyramidal neurons (overactivation-induced inactivation); and that are exacerbated by psychostimulants, and *vice versa* (Wayman et al., 2016; Wayman et al. 2015; Wayman et al. 2012).

In the present study, we used HIV-1 Tg and non-Tg rats (4∼6-week-old, equivalent to the teenage humans) with Meth. Rats received daily repeated subcutaneous (s.c.) injections of Meth for consecutive 5 days, followed by a 3∼4-day withdrawal period. We then utilized electrophysiological approaches (whole-cell patch-clamping) to evaluate the dysfunction of astrocytes in affecting K^+^ homeostasis the mPFC. Because cortical astrocytes are “non-excitable” and do not generate action potentials (firing) in response to excitatory inputs or stimuli, we evaluated their dysfunction by determining the alterations in the resting membrane potential (RMP), as well as inflowing and outflowing K^+^ currents mediated by different subtypes of astrocytic K^+^ channels. This study provides insights into how Meth and/or neuroHIV alter the dynamic activity of cortical astrocytes, their influence on cortical neuron excitability via mediating the K^+^ buffering/homeostasis, and the broader integrity of the mPFC. The current study also defined the potential mechanisms by which combined Meth abuse and neuroHIV disturb the functional activity of live mPFC astrocytes in the brain.

## 2 Methods and materials

### 2.1 Animals

Male F344 non-Tg and HIV-1 Tg rats were breed and group-housed at the Rush University Medical Center animal facility on a 12-h light/dark cycle. Food and water were available *ad libitum.* Animal care and use procedures were conducted in accordance with NIH, USDA and institutional guidelines, and approved by the Institutional Animal Care. Animals were used for experiments at the age of 4–5 weeks (wk).

### 2.2 Brain slice preparation and whole-cell patch-clamp recording in brain slices

Brain slices were prepared as described previously (Chen et al., 2019; Chen et al., 2025; Khodr et al., 2018). In brief, under deep anesthesia with 3% isoflurane with a 2L/min oxygen flow rate, the rats were transcardially perfused with ice-cold solution (in mM: 248 sucrose, 2.9 KCl, 2 MgSO_4_, 1.25 NaH_2_PO_4_, 26 NaHCO3, 0.1 CaCl_2_, and 10 glucose; pH 7.4–7.45) containing 3 mM kynurenic acid and 1 mM ascorbic acid. Brain slices (250 µm coronal sections) containing the mPFC were sectioned and incubated in oxygenated (95% O_2_/5% CO_2_) artificial cerebrospinal fluid (aCSF) consisted of (in mM: 125 NaCl, 2.5 KCl, 25 NaHCO_3_, 1.25 NaH_2_PO_4_, 1 MgCl_2_, 2 CaCl_2_, and 15 glucose; pH 7.4–7.45). After at least 1-h incubation, slices were transferred to a recording chamber perfused with oxygenated aCSF for recording. Nikon ECLIPSE E600FN microscope was used to visually identify astrocytes from the layer IV-V of the mPFC.

Whole-cell voltage-clamping approach was performed to access functional activity of different subtypes of K^+^ channels (e.g., voltage-gated, inwardly rectifying and K_2P_ channels and their alike) in mPFC astrocytes. Glass electrodes (6–10 MΩ) were pulled from borosilicate pipettes using a horizontal pipette puller P-97 (Sutter Instruments Co. Novato, CA) and then filled with an internal solution (in mM: 120 K-gluconate, 20 KCl, 0.1 EGTA, 2 MgCl_2_, 10 HEPES, 3 Na_2_ATP, 0.3 Na_2_GTP; pH: 7.3–7.35; with 270–285mOsm). All astrocytes met the following criteria: (i) smaller diameter (∼10 µm) compared to pyramidal neurons nearby; (ii) the resting membrane potential (RMP) was at −60 mV or more hyperpolarized levels; (iii) there was no action potential (AP) evoked; (iv) the membrane capacity (C_m_) was lower than 20pF; and (v) the serious resistance (R_s_) less than 50 MΩ. The recording protocol included a series of 500 ms current pulses with the intensity ranged from −140 to +100 mV with a 20 mV increment. To access K^+^ channel activity, selective blockers for voltage-gated Na^+^ channels (tetrodotoxin, TTX, 0.5 µM; Abcam) and Ca^2+^ channels (cadmium, Cd^2+^, 200 µM; Sigma) were added in aCSF, which were perfused for at least 10 min prior to electrophysiological assessment.

pClamp 11 software (Molecular Devices, Sunnyvale, CA) was used for acquisition and data analysis. All the K^+^ currents recorded were measured at the steady-state time point 10 ms prior to the end of each step. The current density (pA/pF) was calculated *via* dividing the actual current magnitude by membrane capacitance (C_m_) and compared among astrocytes between non-Tg and HIV-1 Tg rats following different pretreatments. Input resistance (R_in_) was calculated as 10 mV/Δ*I*, while Δ/*=* the difference between the steady state K^+^ currents at the two voltage-steps encompassing 0 pA.

### 2.3 Drug application

Meth was obtained from NIDA Reagent Program and dissolved in saline (SAL) to make 100 mM or 5 mg/mL stock solution. All other chemicals were purchased from Sigma-Aldrich (St. Louis, MO), unless otherwise specified.

To access the effects of Meth *in vitro* on altering the functional activity of K^+^ channels in live, dynamic mPFC astrocytes, Meth with different final concentrations (0, 20, 100 µM) were perfused in the bath using a continuous perfusion system. The assessment was performed after at least 10 min perfusion of each drug concentration.

To access the effects of Meth *in vivo* on functional activity of astrocytic K^+^ channels, we treated rats with daily injections of Meth (5 mg/kg/day s.c.) for 5 days (d), and then killed them after a 3d withdrawal for electrophysiological evaluation. SAL was used as vehicle control for Meth. In another subgroup, rats also received combined daily s.c. injections of EPPTB (a selective trace amine-associated receptor 1 (TAAR1) antagonist, 10 mg/kg, Tocris Biosciences, Bristol, United Kingdom) 15 min prior to Meth or SAL pretreatment. 35% DMSO mixed with 60% PEG400 + 40% SAL were used as a solvent for EPPTB and a vehicle (Veh) control.

### 2.4 Statistical analysis

Data was analyzed using GraphPad Prism 8 (GraphPad Software Inc., La Jolla, CA). The current-voltage relationships (I-V curves) were compared using Two-way rmANOVA with current as repeated factor, followed by Turkey’s (acute Meth experiment) or Sidak’s (daily repeated Meth and/or EPPTB experiments) *post hoc* test. In acute experiments, both voltage and treatment were considered as repeated factors, while in daily repeated Meth exposure and EPPTB pretreatments experiments, only voltage is considered as a repeated factor. Alterations in the resting membrane properties were compared using Two-way ANOVA followed with Bonferroni’s (acute Meth experiment) or Sidak’s (daily repeated Meth and/or EPPTB exposure experiments) multiple comparison test. Data was presented as mean ± SE. Outlier(s) that defined as more than 2-fold the standard deviation from the mean was excluded from data analysis. Statistical significance was generally set at *p* ≤ 0.05.

## 3 Results

### 3.1 Either acute meth exposure *in vitro* or neuroHIV depolarized RMP in mPFC astrocytes, but such alterations were not additivea

**FIGURE 1:**
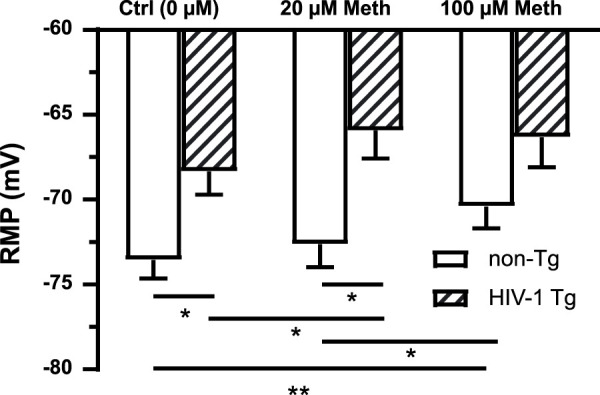
Both acute Meth exposure *in vitro* and neuroHIV depolarized RMP in mPFC astrocytes, but such alterations were not additive. RMP of mPFC astrocytes was depolarized in response to acute Meth *in vitro* (20 and 100 µM) and neuroHIV modeled in HIV-1 Tg rats (n = 14 neurons for non-Tg groups, n = 12 for HIV-1 Tg groups; Genotype effect: F_(1,24)_ = 8.084, *p* = 0.0090; Meth effect: F_(2,48)_ = 9.175, *p* = 0.0004. Bonferroni *post hoc* test: *^,^**p < 0.05, 0.01), suggesting that the mechanism(s) by which acute Meth and neuroHIV disturbed K_2P_/K_2P_-like channels could be similar or interactive.

Cellular RMP was mainly mediated by K_2P_ channel activation. To determine the effects of acute Meth *in vitro* and neuroHIV on astrocyte membrane, we assessed changes in astrocytic RMP in response to acute Meth or in neruoHIV rats. As shown in Figure 1, RMP was significantly depolarized among astrocytes in HIV-1 Tg rats compared to those in non-Tg rats (n = 14/each for non−Tg group, n = 12 for HIV−1 Tg groups; Genotype effect: *p* = 0.0090; Meth effect: *p* = 0.0004; interaction: *p* = 0.1091). Acute Meth *in vitro* also significantly depolarized RMP in mPFC astrocytes from both non-Tg and HIV-1 Tg in a dose dependent manner. However, there was no significant difference between non-Tg and HIV-1 Tg at the highest dose (100 µM) of Meth. These results suggest that acute Meth- and neuroHIV-induced dysfunction of K_2P_/K_2P_−like channels might not be additive.

### 3.2 Acute meth and neuroHIV suppressed K^+^ efflux from mPFC astrocytes, and the greatest reduction occurred following combined exposure to both

**FIGURE 2:**
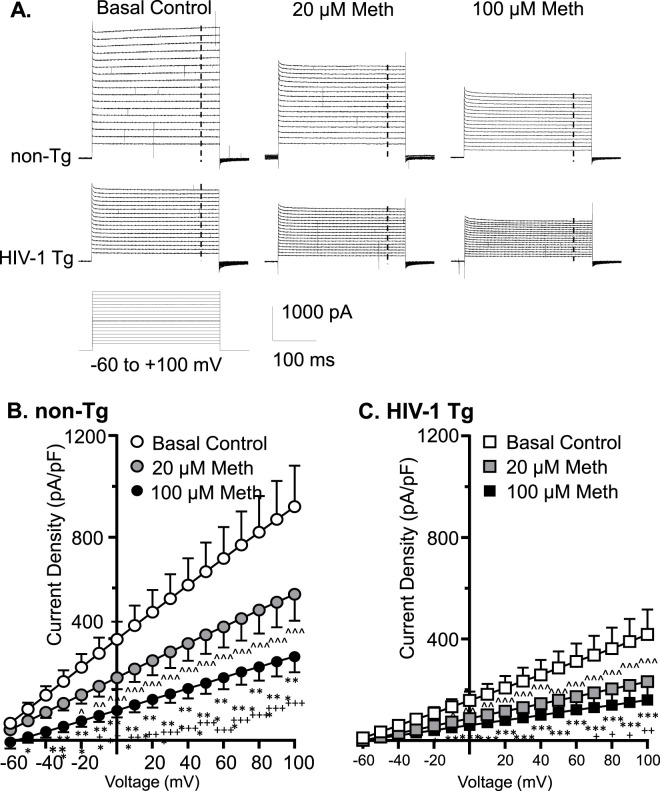
Acute Meth and neuroHIV suppressed K^+^ efflux from mPFC astrocytes, and the greatest reduction occurred following combined exposure to both. **(A)** Sample traces show the voltage-sensitive *I*Kv in mPFC astrocytes during membrane depolarization, with or without acute exposure to Meth in bath (0, 20, and 100 µM), from non−Tg and HIV−1 Tg rats. The vertical dashed lines indicate the time points at which astrocytic K^+^ effluxes we assessed. **(B)** Acute Meth induced a significant decrease in the density of *I*_Kv_ mediated by K_v_ (and K_v_-like) channels in mPFC astrocytes among non−Tg rats (n = 14/ea. Meth effect: F (2, 26) = 8.086, *p* = 0.0019; interaction: F (32, 416) = 8.110, *p <* 0.0001. Tukey’s *post hoc* test: 100 µM Meth vs. basal control 0 μM: *^,^**^,^***p < 0.05, 0.01, 0.001; 20 µM Meth vs. baseline: ^∧,∧∧,∧∧∧^ *p*<0.05, 0.01, 0.001; 100 µM Meth vs. 20 µM Meth: ^+,++,+++^ *p* < 0.05, 0.01, 0.001). **(C)** The density of *I*_Kv_ from astrocytes was also reduced in HIV−1 Tg rats; and that was exacerbated by acute Meth, and *vice versa* (n = 12/ea. Meth effect: F (2,22)=5.712 *p =* 0.0100; interaction: F(32,352) = 5.661, *p* < 0.0001. Tukey’s *post hoc* test: 100 µM Meth vs. basal control 0 μM: *^,^**^,^*** *p*<0.05, 0.01, 0.001; 20 µM Meth vs. baseline: ^∧,∧∧,∧∧∧^ *p* < 0.05, 0.01, 0.001; 100 µM Meth vs. 20 µM Meth: ^+^ *p* < 0.05).

Voltage-gated K^+^ (K_v_) channels, including, but not limited to, the delayed rectifier K^+^ channels and their alike, play a key role in the functional activity of cortical astrocytes via regulating the extracellular K^+^ homeostasis by conducting K^+^ efflux. To examine the effects of acute Meth *in vitro* (20 or 100 µM) on K_v_/K_v_-like channels, we assessed K^+^ currents (*I*Kv) mediated by these K^+^ channels in mPFC astrocytes. We found that cortical astrocyte displayed a large voltage-dependent K^+^ efflux as shown among basal control astrocytes in non-Tg rats (Figure 2A). But acute exposure to Meth (20, 100 µM) induced a significant reduction in *I*_Kv_ among mPFC astrocytes in both non−Tg (n = 14/ea; Meth effect: *p =* 0.0019; voltage effect: *p <* 0.0001; interaction: *p <* 0.0001) (Figures 2A,B) and HIV−1 Tg rats (n = 12/ea; Meth effect: *p =* 0.010; voltage effect: *p* < 0.001; interaction: *p* < 0.001) (Figures 2A,C).

We also fund that voltage-sensitive astrocytic *I*Kv was significantly decreased in HIV-1 Tg rats compared to non-Tg rats, while such reduction was exacerbated by acute Meth, and *vice versa* (Baseline-non-Tg vs. Baseline−HIV-1 Tg: genotype effect: *p =* 0.0106; voltage effect: *p <* 0.0001; interaction: *p <* 0.0001; 20 µM Meth/non− Tg vs. 20 µM Meth/HIV−1 Tg: genotype effect: *p =* 0.0075; voltage effect: *p <* 0.0001; interaction: *p <* 0.0001; 100 µM Meth/non−Tg vs. 100 µM Meth/HIV−1 Tg: genotype effect: *p =* 0.0186; voltage effect: *p <* 0.0001; interaction: *p <* 0.0001) (Figures 2B,C). These results reveal that acute Meth− and neuroHIV-induced RMP dysregulation mediated by K_v_/K_v_-like channels was addictive.

### 3.3 Acute meth and neuroHIV also diminished K^+^ influx to mPFC astrocytes, and the maximal reduction also occurred following combined exposure

**FIGURE 3:**
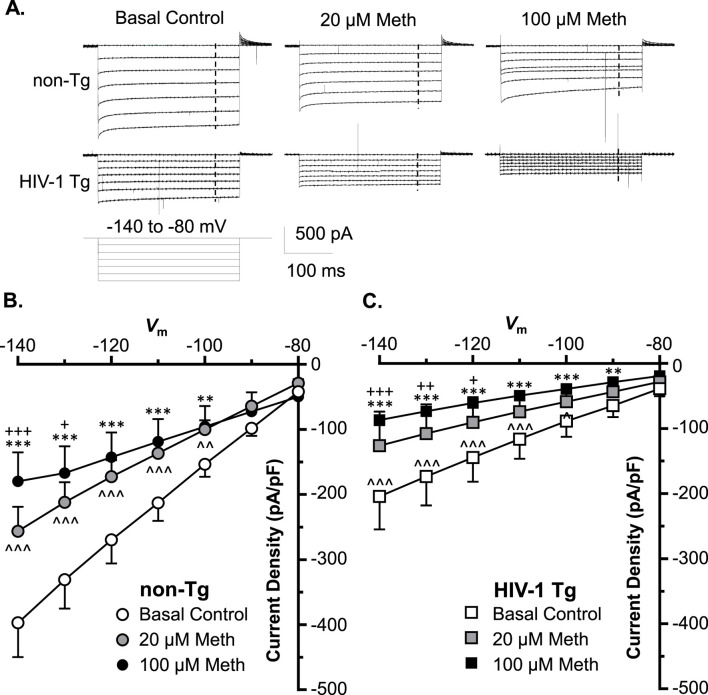
Acute Meth and neuroHIV also diminished K^+^ influx to mPFC astrocytes; and the maximal reduction also occurred following combined exposure. **(A)** Sample traces display the K^+^ influx to mPFC astrocytes (*I*_Kir_, mediated by inwardly rectifying K_ir_ and K_ir_-like channels), before and after acute exposure to Meth (0, 20, and 100 µM, in bath) in the brain slices of non−Tg and HIV−1 Tg rats. The vertical dashed lines indicated the time points, at which the astrocytic K^+^ influxes were measured during membrane hyperpolarization. **(B)** Acute Meth *in vitro* significantly suppressed the density of *I*_Kir_ among mPFC astrocytes in non−Tg rats (n = 14/ea. Meth effect: F(2,26) = 5.008, *p =* 0.0145; interaction: F(12,156) = 10.60, *p <* 0.0001. Tukey’s *post hoc* test: 100 µM Meth vs. baseline: **^,^***p < 0.01, 0.001; 20 µM Meth vs. baseline: ^∧∧,∧∧∧^ *p* < 0.01, 0.001; 100 µM Meth vs. 20 µM Meth: ^+,+++^ *p* < 0.05, 0.001). **(C)** Astrocytic K^+^ influx was also significantly reduced in HIV−1 Tg rats, which was worsened by acute Meth, and *vice versa* (n = 12/ea. Meth effect: F(2,22)=5.060, *p* = 0.0156; interaction: F(12,132) = 5.702, *p* < 0.0001. Tukey’s *post hoc* test: 100 µM Meth vs. baseline: **^,^***p < 0.01, 0.001; 20 µM Meth vs. baseline: ^∧∧∧^ *p* < 0.001; 100 µM Meth vs. 20 µM Meth: ^+,++,+++^ *p* < 0.05, 0.01, 0.001).

To exam the effects of acute Meth on astrocytic K^+^ influx, we assessed inflowing *I*K mediated by inwardly rectifying K^+^ (K_ir_) channels. We found that astrocytes displayed a smaller *I*_Kir_ (compared to *I*_Kv_), which was mainly mediated by K_ir_/K_ir_-like channels in response to *V*_m_ hyperpolarization in non−Tg rats (basal control, V_h_ = −140 to −80 mV levels) (Figure 3A). These K^+^ channels were activated at *V*_m_ levels more hyperpolarized than −60 mV. We found that both 20 and 100 µM acute Meth in bath significantly suppressed *I*_Kir_ when *V*_m_ was hyperpolarized from −140 to −100 mV in cortical astrocyte in non−Tg rats (n = 14/ea. Meth effect: *p =* 0.0145; voltage effect: *p* < 0.0001; interaction: *p <* 0.0001) (Figures 3A,B).

We also detected that astrocytic *I*_*Kir*_ was also reduced significantly in HIV-1 Tg rats compared to non-Tg rats, while this reduction was exacerbated by acute Meth, and *vice versa* (Baseline-non-Tg vs. Baseline-HIV-1 Tg: genotype effect: *p* = 0.0283; voltage effect: *p* < 0.0001; interaction: *p* < 0.0001; 20 µM Meth non−Tg vs. 20 µM Meth HIV−1 Tg: genotype effect: *p* = 0.0371; voltage effect: *p* < 0.0001; interaction: *p* < 0.0001; 100 µM Meth/non− Tg vs. 100 µM Meth/HIV−1 Tg: genotype effect: *p* = 0.0927; voltage effect: *p* < 0.0001; interaction: *p* < 0.0001) (Figures 3B,C). These findings indicate that Meth and neuroHIV-induced K_ir_/K_ir_-like channel dysfunctions are addictive.

### 3.4 Daily repeated meth administration caused RMP depolarization in mPFC astrocytes of non-Tg rats and that was reversed by blocking TAAR1; but meth induced no notable change in astrocytic RMP of HIV-1 Tg rats

**FIGURE 4:**
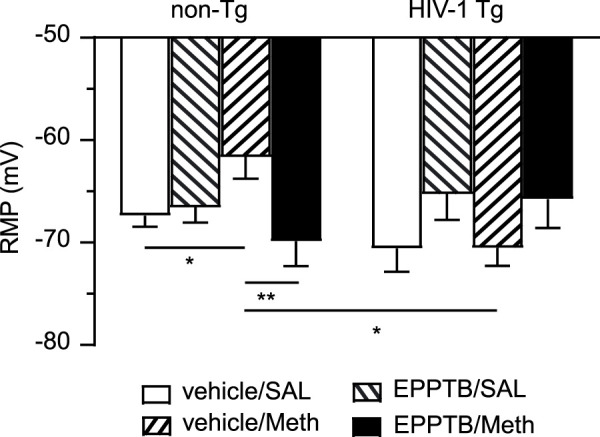
Daily repeated Meth exposure caused RMP depolarization in mPFC astrocytes in non-Tg rats and that was reversed by blocking TAAR1; but Meth induced no notable changes in astrocytic RMP of HIV-1 Tg rats. This chronic effect of Meth was abolished via blocking TAAR1-coupled signaling pathway by a selective TAAR1 antagonist (EPPTB); but EPPTB *per se* did not affect astrocytic RMP, regardless of genotype or pretreatment. There was no significant RMP depolarization among mPFC astrocytes in HIV-1 Tg rats, with or without subchronic Meth (non-Tg rats: vehicle/SAL, EPPTB/SAL, vehicle/Meth, EPPTB/Meth: n = 11,12, 10 vs. 11; EPPTB effect: F(1,40) = 6.246, *p* = 0.0167; interaction: F(1,40) = 8.702, *p* = 0.0053; HIV-1 Tg rats: vehicle/SAL, EPPTB/SAL, vehicle/Meth, EPPTB/Meth: n = 12, 13, 16 vs. 12; EPPTB effect: F(1,49) = 4.624, *p* = 0.0365. With Tukey’s *post hoc* test: *^,^**p < 0.05, 0.01). Combined Meth exposure induced significant hyperpolarization of RMP in HIV-1 rats compared to Meth-exposed non-Tg rats (interaction: F(1,45) = 10.01, *p* = 0.0028. With Tukey’s *post hoc* test: *p < 0.05).

Meth is a potent agonist for TAAR1, and in our previous study we demonstrated that antagonizing TAAR1 with EPPTB *in vitro* abolished acute Meth-induced suppression of *I*_Kv_ and RMP depolarization in human fetal astrocyte (Dave et al., 2019). To determine the effect of TAAR1 blockade on Meth-induced changes in astrocytes, we evaluated the effect of chronic EPPTB *in vivo* on daily repeated Meth-induced K^+^ channel dysfunction in mature astrocytes in the rat brain. We found that there was no significant change in RMP of mPFC astrocytes in non-Tg rats in response to EPPTB treatment, but repeated Meth exposure *in vivo* induced significant depolarization of RMP (which was primarily mediated by K_2P_/K_2P_−like channels) among mPFC astrocytes in non-Tg rats. However, Meth-induced RMP depolarization was completely abolished by combined daily EPPTB treatment in non-Tg rats (vehicle/SAL, EPPTB/SAL, vehicle/Meth, EPPTB/Meth: n = 11,12, 10 vs. 11; EPPTB effect: *p* = 0.0167; Meth effect: *p* = 0.2897; interaction: *p* = 0.0053) (Figure 4). This result indicates that blockade of TAAR1 abolishes Meth effects on interrupting astrocytic K2P/K2P−like channels).

In contrast, we also found that repeated EPPTB pretreatment affected RMP among mPFC astrocytes in HIV-1 Tg rats. Thus, Meth induced no change in astrocytic RMP among HIV-1 Tg rats (vehicle/SAL/HIV-1 Tg, EPPTB/SAL/HIV-1 Tg, vehicle/Meth/HIV-1 Tg, EPPTB/Meth/HIV-1 Tg: n = 12, 13, 16 vs. 12; EPPTB effect: *p* = 0.0365; Meth effect: *p* = 0.9272; interaction: *p* = 0.9124). Further, there was also no significance in RMP after a *post hoc* test in EPPTB-treated HIV-1 Tg rats. These results suggest that the effects of EPPTB, Meth, and neuroHIV (which alter astrocytic RMP individually) might have been reconciled or collapsed when they were combined, regardless of type of pretreatment (Figure 4). Alternatively, the difference detected in RMP of mPFC astrocytes could occur among vehicle/SAL + vehicle/Meth rats and EPPTB/SAL + EPPTB/Meth rats.

We also conducted a cross comparison between non-Tg and HIV-1 Tg rats, showing that there was no significant difference in RMP between SAL/EPPTB-pretreated non-Tg and HIV-1 Tg rats (vehicle/SAL/non-Tg, EPPTB/SAL/non-Tg vs. vehicle/SAL/HIV-1 Tg, EPPTB/SAL/HIV−1 Tg: EPPTB effect: *p* = 0.1363; HIV−1 effect: *p* = 0.6402; interaction: *p* = 0.2675) (Figure 4). However, combined exposure to Meth and neuroHIV (i.e., vehicle/Meth/HIV-1 Tg rats) caused hyperpolarization of RMP in mPFC astrocytes compared to that in vehicle/Meth-pretreated non-Tg rats (vehicle/Meth/non−Tg vs. vehicle/Meth/HIV−1 Tg: EPPTB effect: *p* = 0.2969; HIV−1 effect: *p* = 0.1862; interaction: *p* = 0.0028) (Figure 4). No difference was found in RMP between EPPTB/Meth/non-Tg rats and EPPTB/Meth/HIV-1 Tg rats, indicating RMP depolarization in HIV-1 Tg rats followed concurrent EPPTB/Meth pretreatment compared to those in EPPTB/Meth/non-Tg rats (Figure 4). Together, these findings suggest that persisting exposure to EPPTB, Meth, and neuroHIV could trigger interactive mechanism(s) that could uniquely affect astrocytic RMP.

### 3.5 Both K^+^ efflux and influx were diminished in astrocytes following exposure to meth and/or neuroHIV, while the greatest reduction also occurred in response to combined Meth/neuroHIV exposure

**FIGURE 5:**
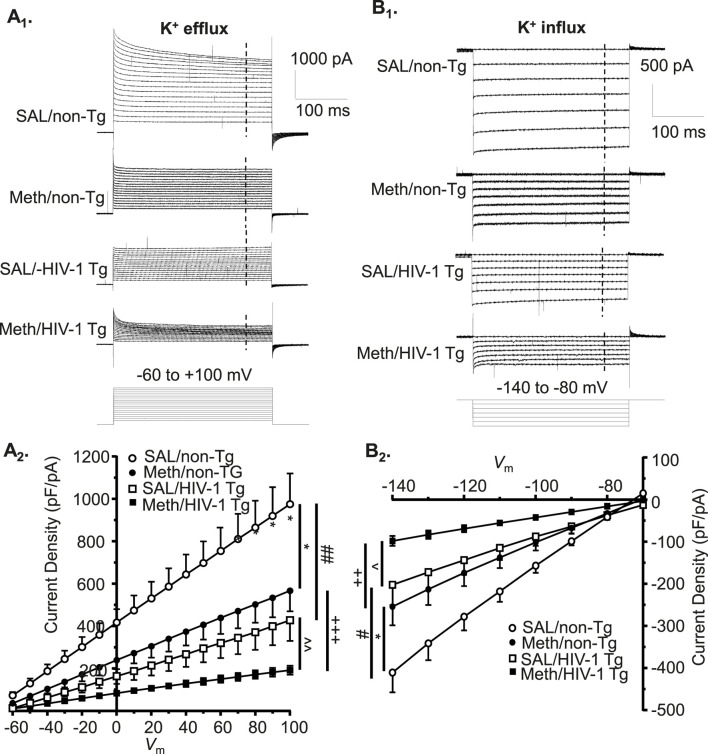
Both K^+^ efflux and influx were diminished in astrocytes following exposure to Meth and/or neuroHIV, while the greatest reduction also occurred in response to combined Meth/neuroHIV exposure. **(A1)** *.* The sample traces of voltage-sensitive K^+^ efflux (*I*_kv_) from astrocytes, with or without Meth exposure *in vivo*, in non-Tg or HIV-1 Tg rats. The vertical dashed lines indicated the time points at which the currents were measured. **(A2)** The current-voltage (I–V) relationships display that the density of outflowing K^+^ currents (*I*_Kv_) were significantly reduced in response to daily repeated Meth or neuroHIV; but the greatest reduction occurred in Meth-pretreated HIV-1 Tg rats (SAL/non-Tg vs. Meth/non-Tg: n = 17 vs. 16; Meth effect: F_(1,31)_ = 5.359, *p* = 0.0274; interaction: F_(16,496)_ = 5.446, *p* < 0.0001. Shown as *p < 0.05; SAL/HIV-1 Tg vs. Meth/HIV-1 Tg: n = 15 vs. 20; Meth effect: F_(1,32)_ = 7.523, *p* = 0.0099; interaction: F_(16,512)_ = 8.972, *p* < 0.0001. As shown as ^∧∧^ *p <* 0.01; SAL/non-Tg vs. SAL/HIV-1 Tg: Genotype effect: F_(1,28)_ = 9.694, *p* = 0.0042; interaction: F_(16,448)_ = 8.346, *p* < 0.0001. As shown as ^##^ *p <* 0.01; Meth/non-Tg vs. Meth/HIV-1 Tg: Genotype effect: F_(1,35)_ = 16.90, *p* = 0.0002; interaction: F_(16,560)_ = 18.47, *p* < 0.0001. Shown as ^+++^ *p <* 0.001). **(B1)** The sample traces of outflowing *I*_Kv_ in astrocytes from non-Tg or HIV-1 Tg rats, with or without Meth exposure. **(B2)** The *I-V* curves show that the density of inflowing *I*_Kir_ was also significantly diminished in the context of Meth abuse or neuroHIV; but the maximum reduction appeared after combined exposure to daily repeated Meth treatments and neuroHIV (SAL/non-Tg vs. Meth/non-Tg: n = 17 vs. 16; Meth effect: F(1,31) = 5.107, *p* = 0.0310; interaction: F(7,217) = 5.776, *p* < 0.0001. Shown as *p < 0.05; SAL/HIV-1 Tg vs. Meth/HIV-1 Tg: n = 13 vs. 21; Meth effect: F(1,32) = 6.323, *p* = 0.0171; interaction: F(7,224) = 6.383, *p* < 0.0001. Shown as ^ *p <* 0.05; SAL/non-Tg vs. SAL/HIV-1 Tg: Meth effect: F(1,28) = 6.954, *p* = 0.0135; interaction: F(7,196) = 10.26, *p* < 0.0001. Shown as ^#^ *p <* 0.05; Meth/non-Tg vs. Meth/HIV-1 Tg: Meth effect: F(1,35) = 12.47, *p* = 0.0012; interaction: F(7,245) = 15.40, *p* < 0.0001. Shown as ^++^ *p <* 0.01).

To exam the impact of Meth *in vivo* and neuroHIV on K^+^ homeostasis mediated astrocytes, we assessed *I*_K_ mediated by voltage-gated K^+^ (K_v_) channels. We found that either daily repeated Meth or neuroHIV modeled in HIV-1 Tg rats induced significant reduction in outflowing K^+^ currents (*I*_Kv_); but the greatest reduction occurred following combined exposure (SAL/non−Tg vs. Meth/non−Tg: n = 17 vs. 16, Meth effect: *p* = 0.0274; voltage effect: *p* < 0.0001; interaction: *p* < 0.0001. SAL/HIV−1 Tg vs. Meth/HIV−1 Tg: n = 15 vs. 20; Meth effect: *p* = 0.0099; voltage effect: *p* < 0.0001; interaction: *p* < 0.0001. SAL/non−Tg vs. SAL/HIV−1 Tg: Genotype effect: *p* = 0.0042; voltage effect: *p* < 0.0001; interaction: *p* < 0.0001. Meth/non−Tg vs. Meth/HIV−1 Tg: Genotype effect: *p* = 0.0002; voltage effect: *p* < 0.0001; interaction: *p* < 0.0001) (Figures 5A1,A2).

Similarly, daily repeated Meth or neuroHIV also induced significant decrease in inflowing K^+^ currents (I_Kir_), and the greatest effect was also found in response to the combined exposure (SAL/non−Tg vs. Meth/non−Tg: n = 17 vs. 16; Meth effect: *p* = 0.0310; voltage effect: *p* < 0.0001; interaction: *p* < 0.0001. SAL/HIV−1 Tg vs. Meth/HIV−1 Tg: n = 13 vs. 21; Meth effect: *p* = 0.0171; voltage effect: *p* < 0.0001; interaction: *p* < 0.0001. SAL/non−Tg vs. SAL/HIV−1 Tg: Meth effect: *p* = 0.0135; voltage effect: *p* < 0.0001; interaction: *p* < 0.0001. Meth/non− Tg vs. Meth/HIV−1 Tg: Meth effect: *p* = 0.0012; voltage effect: *p* < 0.0001; interaction: *p* < 0.0001) (Figures 5B1,B2). Together, these results demonstrate that Meth *in vivo* worsened neuroHIV-induced astrocytic Kv/Kir channel dysfunction, and *vice versa*.

### 3.6 Daily repeated meth-, but not neuroHIV-induced reduction in astrocytic K^+^ efflux was abolished by blocking TAAR1

**FIGURE 6:**
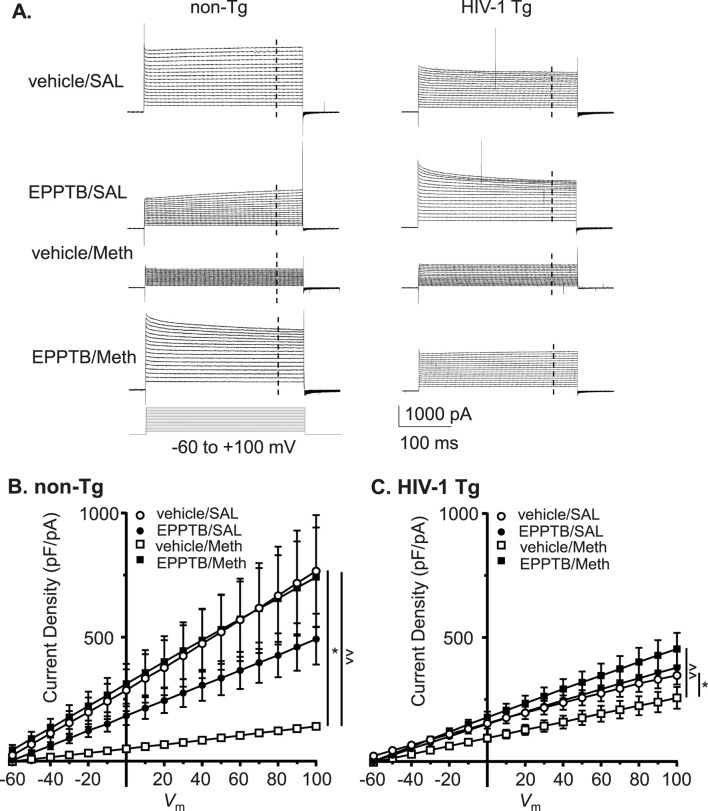
Daily repeated Meth-, but not neuroHIV-induced K^+^ efflux reduction was abolished by blocking TAAR1 in mPFC astrocytes. **(A)** The sample traces of outflowing *I*_Kv_ from mPFC astrocytes in non-Tg rats (left panel) and HIV-1 Tg rats (right panel), with or without daily repeated administration of Meth and/or the TAAR1 antagonist EPPTB (dissolved in the vehicle, DMSO). The vertical dashed lines indicated the time points at which we measured the currents. **(B)** Daily repeated Meth-induced reduction in the density of outflowing *I*_Kv_ from mPFC astrocytes was completely abolished by blocking TARR1 in non-Tg rats (vehicle/SAL/non-Tg, vehicle/Meth/non-Tg, EPPTB/SAL/non-Tg vs. EPPTB/Meth/non-Tg: n = 11, 10, 12 vs. 11. vehicle/SAL/non-Tg vs. vehicle/Meth/non-Tg: Meth effect: F_(1,19)_ = 6.992, *p* = 0.0160; interaction: F_(16,304)_ = 6.991, *p* < 0.0001; shown as *p < 0.05; vehicle/Meth/non-Tg vs. EPPTB/Meth/non-Tg: EPPTB effect: F_(1,19)_ = 8.958, *p* = 0.0075; interaction: F_(16,304)_ = 7.762, *p* < 0.0001; shown as ^∧∧^ *p <* 0.01; Vehicle/SAL/non-Tg vs. EPPTB/SAL/non-Tg: *p* > 0.05; EPPTB/SAL/non-Tg vs. EPPTB/Meth/non-Tg: *p* > 0.05). **(C)** Subchronic Meth-induced decrease in outflowing *I*_Kv_ from astrocytes was also reversed by blocking TARR1 (with EPPTB) in HIV-1 Tg rats (vehicle/SAL/HIV-1 Tg, vehicle/Meth/HIV-1 Tg, EPPTB/SAL/HIV-1 Tg vs. EPPTB/Met/HIV-1 Tg: n = 12, 12, 12 vs. 11. vehicle/SAL/HIV-1 Tg vs. vehicle/Meth/HIV-1 Tg: Meth effect: F_(1,22)_ = 4.469, *p* = 0.0461; shown as *p < 0.05; vehicle/Meth/HIV-1 Tg vs. EPPTB/Meth/HIV-1 Tg: EPPTB effect: F_(1,21)_ = 8.336, *p* = 0.0088; interaction: F_(16,336)_ = 5.253, *p* < 0.0001; shown as ^^ *p <* 0.01; vehicle/SAL/HIV-1 Tg vs. EPPTB/SAL/HIV-1 Tg: *p* > 0.05; EPPTB/SAL/HIV-1 Tg vs. EPPTB/Meth/HIV-1 Tg: *p* > 0.05).

To determine the effect of TAAR1 antagonism on Meth and/or neuroHIV-induced dysregulation of astrocytic *I*_Kv_, we evaluated EPPTB’s effect on Meth and/or neuroHIV-induced changes in *I*_Kv_. We found that Meth-induced reduction in outflowing *I*_Kv_ from astrocytes was eliminated by antagonizing TARR1 with repeated EPPTB pretreatments in non-Tg rats (vehicle/SAL/non-Tg, vehicle/Meth/non-Tg, EPPTB/SAL/non-Tg vs. EPPTB/Meth/non-Tg: n = 11,10,12 vs. 11. vehicle/AL/non−Tg vs. vehicle/Meth/non−Tg: Meth effect: *p* = 0.0160; voltage effect: *p* < 0.0001; interaction: *p* < 0.0001; vehicle/Meth/non−Tg vs. EPPTB/Meth/non−Tg: EPPTB effect: *p* = 0.0075; voltage effect: *p* < 0.0001; interaction: *p* < 0.0001) (Figures 6A,B). Meth-induced decrease in *I*_Kv_ from astrocytes was also reversed by blocking TAAR1 with EPPTB pretreatments in HIV-1 Tg rats (vehicle/SAL/HIV-1 Tg, vehicle/Meth/HIV-1 Tg, EPPTB/SAL/HIV-1 Tg vs. EPPTB/Met/HIV-1 Tg: n = 12, 12, 12 vs. 11. vehicle/SAL/HIV-1 Tg vs. vehicle/Meth/HIV−1 Tg: Meth effect: *p* = 0.0461; voltage effect: *p* < 0.0001; interaction: *p* = 0.1658; vehicle/Meth/HIV−1 Tg ^vs.^ EPPTB/Meth/HIV−1 Tg: EPPTB effect: *p* = 0.0088; voltage effect: *p* < 0.0001; interaction: *p* < 0.0001) (Figures 6A,C). Nevertheless, repeated EPPTB treatment alone showed no significant effect on altering *I*_Kv_ in either non−Tg rats (vehicle/SAL/non−Tg vs. EPPTB/SAL/non−Tg: EPPTB effect: *p* = 0.2579; voltage effect: *p* < 0.0001; interaction: *p* = 0.2700) (Figure 6B), or HIV−1 Tg rats (vehicle/SAL/HIV−1 Tg vs. EPPTB/SAL/HIV−1 Tg: EPPTB effect: *p* = 0.9355; voltage effect: *p* < 0.0001; interaction: *p* = 0.987) (Figure 6C). These findings indicate that daily repeated Meth-induced Kv/Kv-like channel dysfunction was mediated via the TAAR1-coupled signaling pathway among cortical astrocytes in the brain.

### 3.7 Antagonizing TAAR1 also abolished meth-induced decrease in inflowing *I*_Kir_ of astrocytes, regardless of genotype

**FIGURE 7:**
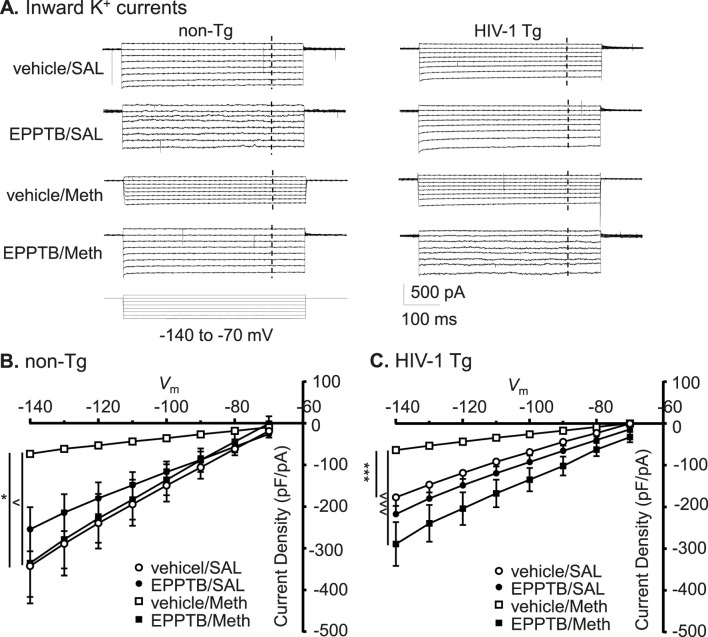
Antagonizing TAAR1 also abolished Meth-induced decrease in inflowing *I*_Kir_ of astrocytes, regardless of genotype. **(A)** The sample traces of inflowing *I*_Kir_ into astrocyte in non-Tg rats (left panel) and HIV-1 Tg rats (right panel), with or without Meth and/or EPPTB. The vertical dashed lines indicated the time points at which the currents were evaluated. **(B)** The I-V relationships show that the density of inflowing *I*_Kir_ to astrocytes were significantly diminished following daily repeated Meth exposure *in vivo*, and this Meth effect was reversed by persistent antagonism of TAAR1 (with EPPTB) in non-Tg rat (vehicle/SAL/non-Tg, EPPTB/SAL/non-Tg, vehicle/Meth/non-Tg vs. EPPTB/Meth/non-Tg: n = 11, 12, 10 vs. 11; vehicle/SAL/non-Tg vs. vehicle/Meth/non-Tg: Meth effect: F_(1,19)_ = 7.904, *p* = 0.0111; interaction: F_(7,133)_ = 8.039, *p* < 0.0001; shown as *p < 0.05; vehicle/Meth/non-Tg vs. EPPTB/Meth/non-Tg: EPPTB effect: F_(1,19)_ = 7.201, *p* = 0.0147; interaction: F_(7,133)_ = 10.53, *p* < 0.0001; shown as ^∧^ *p <* 0.05; EPPTB/SAL/non-Tg vs. EPPTB/Meth/non-Tg: *p* > 0.05; Vehicle/SAL/non-Tg vs. EPPTB/SAL/non-Tg: *p* > 0.05). **(C)** The *I-V* curves show that inflowing *I*_Kir_ was not reduced in HIV-1 Tg rats compared to non-Tg rats, while persisting TAAR1 blockade *in vivo* by repeated EPPTB administrations did not affect such change in *I*_Kir_. However, daily repeated Meth-induced reduction in this K^+^ influx (even with neuroHIV) was blocked by EPPTB (HIV-1 Tg: vehicle/SAL, EPPTB/SAL, vehicle/Meth vs. EPPTB/Meth: n = 12, 12, 13 vs. 11; vehicle/SAL/HIV-1 Tg vs. vehicle/Meth/HIV-1 Tg: Meth effect: F_(1,23)_ = 28.18, *p* < 0.0001; interaction: F_(7,161)_ = 27.88, *p* < 0.0001; shown as ***p < 0.001; vehicle/Meth/HIV-1 Tg vs. EPPTB/Meth/HIV-1 Tg: EPPTB effect: F_(1,22)_ = 19.36, *p* = 0.0002; interaction: F_(7,154)_ = 27.88, *p* < 0.0001; shown as ^∧∧∧^ *p <* 0.001; EPPTB/SAL/HIV-1 Tg vs. EPPTB/Meth/HIV-1 Tg: *p* > 0.05). Inflowing *I*_Kir_ was not significantly affected by pretreatments of EPPTB *per se* in HIV-1 Tg rats (Vehicle/SAL/HIV-1 Tg vs. EPPTB/SAL/HIV-1 Tg: *p* > 0.05).

To define the mechanism underlying the changes in K_ir_ channels, we identified the effects of TAAR1 antagonist on Meth and/or neuroHIV-induced changes in *I*_Kir_ among cortical astrocytes. We found that astrocytic *I*_Kir_ was significantly reduced following Meth *in vivo*, and this Meth effect was reversed by persistent antagonizing TAAR1 with EPPTB in non- Tg rats (vehicle/SAL/non-Tg, EPPTB/SAL/non-Tg, vehicle/Meth/non-Tg vs. EPPTB/Meth/non-Tg: n = 11, 12, 10 vs. 11; vehicle/SAL/non−Tg vs. vehicle/Meth/non−Tg: Meth effect: *p* = 0.0111; voltage effect: *p* < 0.0001; interaction: *p* < 0.0001; shown as *p < 0.05; vehicle/Meth/non−Tg vs. EPPTB/Meth/non−Tg: EPPTB effect: *p* = 0.0147; voltage effect: *p* < 0.0001; interaction: *p* < 0.0001; shown as ^*p* < 0.05; EPPTB/SAL/non−Tg vs. EPPTB/Meth/non−Tg: Meth effect: *p* = 0.6103; voltage effect: *p* < 0.0001; interaction: *p* = 0.2028). In contrast, *I*_Kir_ was not significantly affected by EPPTB alone in non-Tg rats (Vehicle/SAL/non-Tg vs. EPPTB/SAL/non- Tg: EPPTB effect: *p* = 0.4618; voltage effect: *p* < 0.0001; interaction: *p* = 0.4368) (Figures 7A,B).

We also detected that similar results occurred in HIV-1 Tg rats, in which Meth caused a significant decrease in K^+^ influx, and this Meth effect was also eliminated by persisting blockade of TAAR1 with EPPTB (vehicle/SAL/HIV-1 Tg, EPPTB/SAL/HIV-1 Tg, vehicle/Meth/HIV-1 Tg vs. EPPTB/Meth/HIV-1 Tg: n = 12, 12, 13 vs. 11; vehicle/SAL/HIV-1 Tg vs. vehicle/Meth/HIV-1 Tg: Meth effect: *p* < 0.0001; voltage effect: *p* < 0.0001; interaction: *p* < 0.0001; vehicle/Meth/HIV−1 Tg vs. EPPTB/Meth/HIV−1 Tg: EPPTB effect: *p* = 0.0002; voltage effect: *p* < 0.0001; interaction: *p* < 0.0001). Meanwhile, we also found that repeated EPPTB pretreatments alone did not significantly affect inflowing *I*_Kir_ in astrocytes of HIV−1 Tg rats (vehicle/SAL/HIV−1 Tg vs. EPPTB/SAL/HIV−1 Tg: EPPTB effect: *p* = 0.2871; voltage effect: *p* < 0.0001; interaction: *p* = 0.9659) (Figures 7A,C). On the other hand, inflowing *I*_Kir_ in mPFC astrocytes was significantly decreased in HIV-1 Tg rats compared to those in non-Tg rats (Figures 7B,C vehicle/SAL/non-Tg vs. vehicle/SAL/HIV−1 Tg: genotype effect: *p* = 0.0537; voltage effect: *p* < 0.0001; interaction: *p* = 0.0077), while Meth-induced reduction in inflowing *I*_Kir_ was also greater in HIV-1 Tg rats compared to non-Tg rats (vehicle/SAL/non-Tg vs. vehicle/SAL/HIV- 1 Tg: genotype effect: *p* = 0.0339; voltage effect: *p* < 0.0001; interaction: *p* = 0.9947) (Figures 7B,C).

**FIGURE 8:**
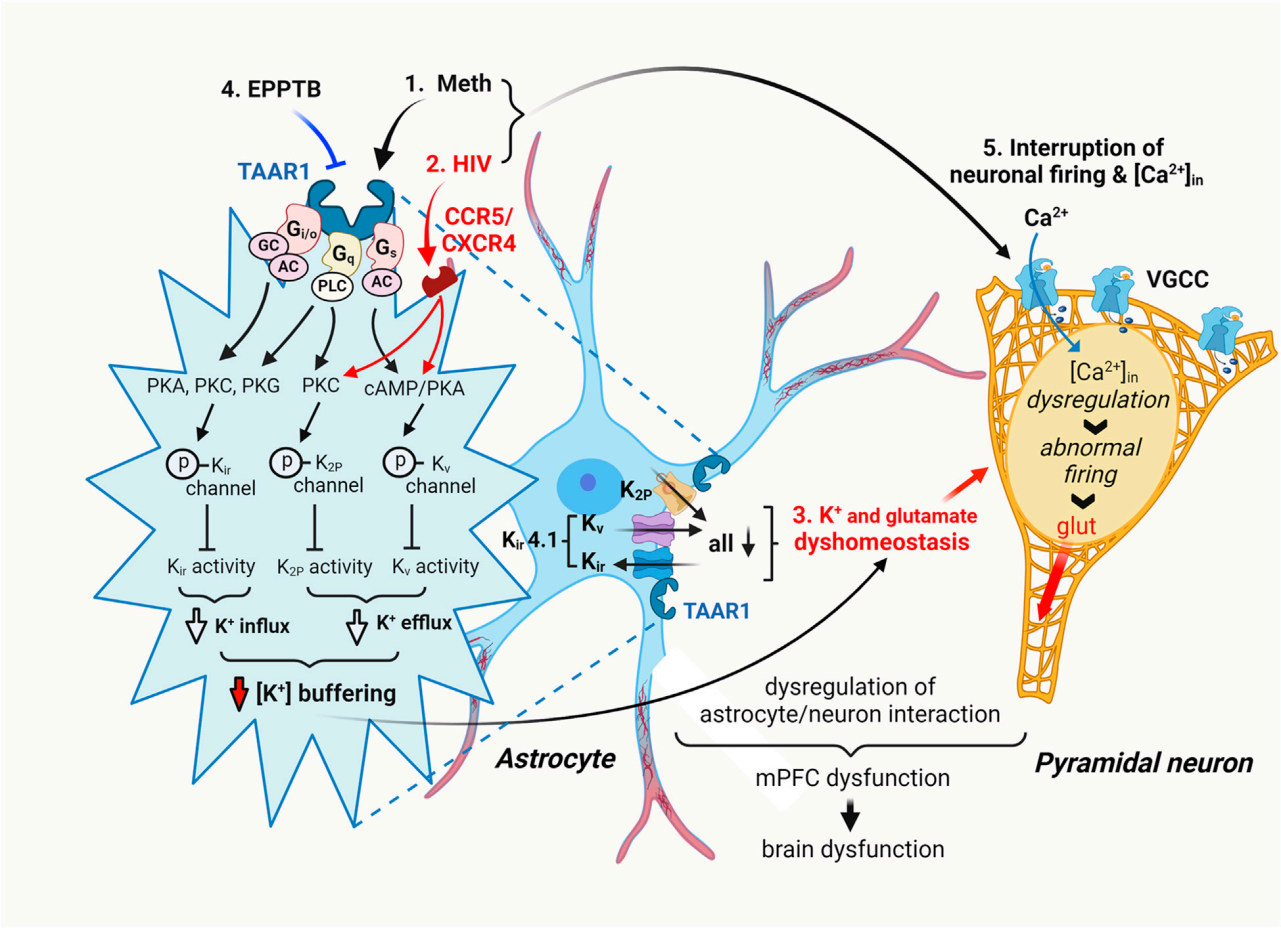
The hypothesized mechanisms underlying Meth- and neuroHIV-induced disruption of astrocyte activity and [K^+^]_e_ homeostasis in the mPFC. Cortical astrocytes play a critical role in supporting normal neuron function by regulating and maintaining extracellular K^+^ homeostasis ([K^+^]_e_) in the brain. Repeated exposure to Meth *in vivo*, with or without withdrawal, suppresses the dynamic function of mPFC astrocytes in regulating/maintaining [K^+^]_e_ homeostasis by disturbing K^+^ efflux/influx through dysfunctional K_v_, K_2P_, and K_ir_ channels and their alike. Meth is an effective inhibitor for catecholamine transporters and a potent agonist for TAAR1 expressed in cortical astrocytes. Thus, it not only blocks reuptake of dopamine, serotonin, and norepinephrine, but also activates the TAAR1/G_s_/cAMP/PKA signaling, as well as the TAAR1/G_i/o_/cAMP/PKA and G_q_/PLC/PKC signaling pathways. These effects of Meth promote PKA, PKC, and PLC-induced phosphorylation of K_v_, K_2P_ and K_ir_ channels, respectively, diminishing their activity in regulating astrocytic K^+^ influx/efflux (a.k.a. K^+^ spatial buffering) and consequently disturbing [K^+^]_e_ homeostasis. Interestingly, cortical astrocytes also express K_ir_4.1 channels (and their alike), which mediate K^+^ influx and efflux in a bidirectional way. This unique effect of K_ir4.1_/K_ir4.1_-like channels is also suppressed by Meth. On the other hand, astrocytic K^+^ channel activity is also diminished in the context of neuroHIV, inducing similar [K^+^]_e_ dyshomeostasis in the mPFC. Meanwhile, astrocytic [K^+^]_e_ dyshomeostasis substantially interrupts reuptake of extracellular glutamate levels ([glut]_e_), thereby disturbing [glut]_e_, and *vice versa*. The astrocyte dysfunction-induced [K^+^]_e_ and [glut]_e_ dyshomeostasis significantly alters the membrane excitability of surrounding cortical pyramidal neurons, inducing hypoactivity and hyperactivity of them, depending on a decrease and increase in [K^+^]_e_/[glut]_e_ levels, respectively. Under these conditions, abnormally increased [K^+^]_e_ inhibits glutamate reuptake, while aberrantly elevated [glut]_e_ also suppresses K^+^ cannel activity. Collectively, these results demonstrate that daily repeated Meth administrations persistently suppress the functional activity of mPFC astrocytes in regulating [K^+^]_e_ (and [glut]_e_) homeostasis in the brain, and this effect is exacerbated in the context of neuroHIV, and *vice versa*. They also suggest that the impact of Meth is mediated by TAAR1/G protein-coupled signaling pathway, while neuroHIV-induced astrocytic K^+^ channel dysfunction is mediated jointly by TAAR1-and CCR5/CXCR4-mediated signaling pathways.

Collectively, these results strongly suggest that Meth-induced dysfunction of K_ir_/K_ir_-like channels in cortical astrocytes was mediated by TAAR1 through its interaction with G protein-coupled signaling pathway. In contrast, the decreased function of astrocytic K_ir_/K_ir_-like channels in HIV−1 Tg rats was regulated by mechanism(s) differing from the TAAR1/G protein-coupled signaling (Figure 8).

## 4 Discussion

The present study characterized the electrophysiological properties and dynamic activity of live mPFC astrocytes in the brain of rats and examined how these properties are significantly affected by acute and daily repeated Meth exposure, *in vitro* and *in vivo*, respectively, with or without the influence of neuroHIV modeled in HIV-1 Tg rats. Specifically, we assessed the alterations in the functional activity of different subtypes of K^+^ channels in mPFC astrocytes that mediate the resting membrane potential (RMP), voltage-gated K^+^ efflux, and inwardly rectifying K^+^ influx, revealing dysfunction of mature cortical astrocytes in the brain.

### 4.1 Acute meth exposure or neuroHIV caused depolarization of RMP in mPFC astrocytes

The current study indicated that both acute Meth and neuroHIV induced RMP depolarization in cortical astrocytes, revealing diminished K_2P_/K_2P_−like channel function. However, there was no additive effect in RMP depolarization following combined Meth (100 µM in bath) and neuroHIV modeled in HIV-1 Tg rats. Because RMP is mediated by K_2P_/K_2P_−like K^+^ channels in all cells (Hille 2001), including cortical astrocytes (Dave et al., 2019), this finding suggests that both acute Meth and neuroHIV inhibit the activity of K_2P_/K_2P_−like channels, causing a decrease in K^+^ efflux and an increase in intracellular K^+^ levels that depolarize astrocytic RMP. The absence of additive effect suggests that the impact of Meth or neuroHIV might have maximally suppressed K_2P_/K_2P_−like channel activity, or they might operate via similar mechanism in disturbing RMP. As expected, this finding is in agreement with our previous study that determined acute effect of Meth on RMP of cultured human fetal astrocytes (Dave et al., 2019).

### 4.2 Acute meth and/or neuroHIV diminished K^+^ efflux and influx in mPFC astrocytes

The current study also demonstrated that acute Meth and neuroHIV induced a significant decrease in voltage-sensitive outward K^+^ currents (K_v_ efflux, or *I*_Kv_) from cortical astrocytes during membrane depolarization; but displayed a maximum decrease in K_v_ efflux in response to combined exposure compared to each individual exposure. Such reduced *I*_Kv_ flowed through dysfunctional K_v_/K_v_-like channels, as we found in cultured human fetal astrocytes (Dave et al., 2019). Meanwhile, the inwardly rectifying K^+^ currents (K^+^ influx, or *I*_Kir_) evoked by membrane hyperpolarization were also diminished by acute Meth or in the context of neuroHIV. Because membrane hyperpolarization-induced K^+^ flux is mainly mediated by inwardly rectifying K^+^ (K_ir_) channels (Hille 2001), this result indicated that activity of both K_v_/K_v_-like and K_ir_/K_ir_-like channels are diminished following exposure to Meth and neuroHIV. Importantly, in contrast to the effects of combined acute Meth and neuroHIV on K_2P_/K_2P_-like channels, the greatest decrease in K^+^ efflux or influx was found in astrocytes following combined exposures. This finding suggests that under the condition of combined acute Meth and neuroHIV, astrocytic K_v_ and K_ir_ channels could experience much severe dysfunction, while the difference in the structures and independence in signaling pathways could underlie the diversity between K_2P_ and K_v_/K_ir_ dysfunction.

### 4.3 Daily repetitive meth persistently depolarized astrocytic RMP; and that was mediated by TAAR1-mediated signaling pathway

Besides acute effect of Meth on astrocytic RMP, the present study further revealed that daily repetitive Meth *in vivo* persistently depolarized RMP in non-Tg control rats, which was prevented by co-administration of the selective TAAR1 antagonist EPPTB. This result was also similar to that we found in human fetal astrocytes in response to acute Meth (Dave et al., 2019). Together, these findings not only reveal a persisting effect of Meth on disturbing cortical astrocyte activity, but also identify the mechanism by which either acute or daily repeated Meth interrupts K_2P_/K_2P_-like channel activity of cortical astrocytes in the brain.

### 4.4 Meth and neuroHIV synergistically disturbed K^+^ homeostasis mediated by astrocytes

Another key finding of the current study was that daily repeated Meth significantly suppressed both K^+^ efflux and influx in astrocytes by inhibiting the activity of K_v_/K_v_-like and K_ir_/K_ir_-like channels; and such Meth effects were exacerbated by neuroHIV, and *vice versa*. While the exact mechanism underlying these changes is not fully understood, the results from the present study indicated that blockade of TAAR1-mediated signaling abolished the effects of daily repeated Meth on astrocytic K^+^ channels. Again, this finding was similar to the effects of acute Meth on these K^+^ channels in cultured human fetal astrocytes (Dave et al., 2019).

However, it is worth noting that despite antagonism of TAAR1, *I*_Kv_ reduction remained in the context of neuroHIV. This finding suggests the involvement of alternative mechanisms, likely via chemokine receptor-mediated pathways. Previous studies identified that CCR5 (C−C chemokine receptor type 5) and CXCR4 (C−X-C chemokine receptor type 4), which are coupled with G proteins (Gs, Gi, Go, or Gq) and mediate PKA, PKC, and PLC activity (Tran and Miller, 2003; Kaul et al., 2001; Dorf et al., 2000; Johansson et al., 2004; Luo et al., 2022), could critically affect astrocyte activity in the context of neuroHIV. These receptors serve as HIV co-receptors and respond to HIV and viral proteins (Yandrapally et al., 2021; Lee et al., 2003). Interestingly, TAAR1 blockade did eliminate the combined effects of daily repeated Meth and neuroHIV on K_ir_/K_ir_-like channel-mediated *I*_Kir_, suggesting that there may be multiple and merged signaling pathways (e.g., mediated by certain G proteins that also coupled with chemokine receptors) involved in disruption of astrocytic K^+^ channel function (Figure 8).

### 4.5 The mechanisms underlying K^+^ channel dysfunction and K^+^ dyshomeostasis in meth and neuroHIV-altered mPFC

Our findings from this study reveal both similarity and complexity in astrocytic K^+^ channel dysfunction induced by daily repeated Meth and/or neuroHIV. Both conditions significantly suppress the activity of K^+^ channels in maintaining and regulating RMP, K^+^ efflux, and K^+^ influx, with maximal suppression of K_v_ and K_ir_ channels (and their alike) occurred during combined exposure. These novel findings strongly suggest both *shared* and *distinct* mechanisms underlying the dysfunction of cortical astrocytes operating through multiple intracellular signaling pathways.

To our knowledge and understanding, these findings provide the first evidence in the field, indicating that daily repeated exposure to Meth persistently suppresses the activity of K_2P_/K_2P_−like, K_v_/K_v_-like, and K_ir_/K_ir_-like channels in mature and dynamically active cortical astrocytes, mainly by activating TAAR1/G protein/PKA, PKC, and/or PLC-coupled signaling pathways (Figure 8). Similarly, neuroHIV also induces comparable astrocyte dysfunction, but probably through both TAAR1-dependent and other independent mechanisms, including, but not limited to, CCR5 and CXCR4-coupled signaling pathways, associated with the involvement of various G proteins (e.g., the stimulatory Gs, inhibitory Gi, Go, or Gq) that also mediate the activity of PKA, PKC (phospholipase A and C, respectively) and PLC (phospholipase C) (Tran and Miller, 2003; Kaul et al., 2001; Dorf et al., 2000; Johansson et al., 2004; Luo et al., 2022). Moreover, the difference between the impact of Meth and neuroHIV on cortical astrocytes also suggests the diversities in the distinct structure and signaling pathways coupled with TAAR1 and chemokine receptors, respectively, which could shed light on the development of new therapeutic strategy for treating MUD and HAND.

Based upon our novel findings and the mechanism of action of TAAR1 antagonist and CCR5 inhibitor (e.g., maraviroc, a FDA-approved anti-HIV medicine that prevents HIV entry into CD4^+^ T cells), we propose that such antagonist/inhibitor may individually or jointly reduce the effects of Meth and neuroHIV on astrocytes and neurons, thereby improving the brain function. In addition, our results also suggest that K^+^ channel activators may attenuate Meth- and neuroHIV-induced K^+^ channel dysfunction, even though the existence of numerous K^+^ channel subtypes could create significant difficulty in selectively activating dysfunctional K^+^ channels. Thus, further research is needed for developing such therapeutic approaches to treat MUD and HAND.

The resulting dysfunction of astrocytic K^+^ channels in the brain could consequently and profoundly alter extracellular K^+^ homeostasis ([K^+^]_e_) in two ways: elevating [K^+^]_e_ when abnormal K^+^ efflux exceeds its influx, thereby significantly increasing [K^+^]_e_ that could induce or promote Meth- and/or neuroHIV-induced hyperactivity of surrounding cortical neurons, leading to neurotoxicity. Or reducing [K^+^]_e_ when disrupted K^+^ influx exceeds its efflux, which would decrease [K^+^]_e_ and induce neuronal hypoactivity, thereby inhibiting cortical neuron activity. Both extracellular K^+^ dyshomeostasis, when that are severe, could cause not only dysfunction, but also injury and even death of astrocytes and neurons in the brain.

Of particular importance among astrocytic K^+^ channels could be the K_ir_4.1 channels, which are exclusively expressed in glial cells (Butt and Kalsi, 2006), but not neurons, in the brain after p15∼p21 (Dave et al., 2019; Kressin et al., 1995; Olsen et al., 2006; Montiel-Herrera and Garcia-Colunga, 2010); and with the highest expression in astrocytes (Olsen et al., 2015). As a key K^+^ channel subtype in astrocytes (Butt and Kalsi, 2006; Djukic et al., 2007; Seifert et al., 2009), K_ir_4.1 channels dynamically regulate [K^+^]_e_ via the mechanism of K^+^ special buffering and K^+^ siphoning (Butt and Kalsi 2006; Kofuji and Newman 2004). Further, these K_ir_4.1 channels are also unique, playing very important role in the CNS, which includes, but not limited to, regulating a bi-directional regulation of K^+^ influx and efflux, RMP, cell volume, and glutamate uptake (Nwaobi et al., 2016). Moreover, there is also a crucial interaction between extracellular K^+^ and glutamate levels in the brain: the activity of astrocytic glutamate transporters to reuptake extracellular glutamate is [K^+^]_o_-dependent, while abnormal increase in [K^+^]_o_, e.g., as a result of decreased activity of K_ir_4.1 channels (Olsen and Sontheimer 2008), inhibits the activity of astrocytes to uptake glutamate, and *vice versa* (Rimmele et al., 2017).

The consequential effects of such extracellular K^+^ and glutamate dyshomeostasis in the brain are critical. Dysfunction of these astrocytic K^+^ channels, including, but not limited to, K_ir_4.1 channels, could abnormally reduce or elevate extracellular K^+^ levels; and therefore, induce neuronal hypoactivity and promote hyperexcitability, respectively. It is worth noting that the latter one could significantly diminish the activity of astrocytic glutamate transporters, aberrantly increasing the extracellular glutamate levels. This combined dyshomeostasis of extracellular K^+^ and glutamate levels could induce, facilitate, or further enhance neurotoxicity in the brain in various neuropathological conditions, including but not limited to MUD, cocaine use disorders, neuroHIV, Alzheimer’s disease, and the comorbidity of them (Hu, 2016; Hu, 2024). Collectively, our novel findings from the current study suggest that chronic exposure to Meth and neuroHIV either individually or jointly suppress the functional activity of K_ir_4.1 channels as well as other subtypes/families of K^+^ channels among mature mPFC astrocytes in the brain, thereby substantially promoting excitotoxicity in dysfunctional mPFC pyramidal neurons nearby (Figure 8). Such disruption of cortical neuron activity could contribute to the decline of function in the brain regions that regulate cognition in HAND and MUD.

Given that both cocaine and Meth are potent psychostimulants with similar but not the same mechanism in interrupting various neurotransmissions (e.g., catecholamines and others), it is possible that repetitive chronic cocaine exposure could induce similar dysfunction of K^+^ channels in cortical astrocytes, which is even founded in neurons (refers to our previous publication, Nasif et al., 2005; JPET, 312:1305-313). Nevertheless, opioids could induce K^+^ channel dysfunction through different mechanisms and signaling pathways.

## 5 Limitation of the present study

There are also some limitations in this present study. For instance, due to the limited resources and scope of this study, we have not evaluated potential alterations in single channel activity; K^+^ channel expression (K_ir_4.1 channels or other K^+^ channel subtypes); concentrations of [K^+^]_e_ and [glut]_e_ in the mPFC; and the effects of specific K^+^ channel activators and selective CCR5/CXCR4 inhibitors on intervening Meth and neuroHIV-induced K^+^ channel dysfunction, respectively. In addition, the mechanism that we proposed regarding the possible involvement of CCR5 and CXCR4 in neuroHIV-induced K^+^ channel dysfunction is hypothetic and needs to be tested further in future study utilizing selective CCR5 and CXCR4 inhibitors. Future investigations focus on addressing these issues could have significant impact on the related fields.

## 6 Summary

The current study demonstrates the impact of acute/daily repeated Meth and neuroHIV on the activity of K^+^ channel subtypes in mature, live mPFC astrocytes in the brain, which differs from the studies of others focusing on demonstrating astrocyte dysregulation of chemokines and cytokines in the immune system. Here we identify dysfunction of astrocytic K^+^ channels that could interrupt K^+^ spatial buffering/siphoning (K^+^ homeostasis) and the interplay of cortical astrocytes with surrounding pyramidal neurons in the mPFC. Consequently, these astrocyte dysfunctions could profoundly alter the excitability of surrounding neurons, initiating and/or enhancing neurotoxicity. Combined exposure to Meth and neuroHIV *in vivo* exacerbates astrocytic K^+^ channel dysfunctions and K^+^ dyshomeostasis compared to that induced by either one alone. Moreover, our novel findings also indicate that Meth-induced astrocytic K^+^ channel dysfunction is mediated through the TAAR1/G proteins/PKA, PKC and PLC-coupled signaling pathways, suggesting that neuroHIV-induced suppression of astrocytic K^+^ channel activity is likely mediated by CCR5 and/or CXCR4, but not by TAAR1s. Importantly, these two signaling pathways could merge in cortical astrocytes to jointly inhibit K^+^ channel activity, disrupting extracellular K^+^ and glutamate homeostasis, and ultimately disturbing the membrane excitability and activity of mPFC pyramidal neurons nearby; and that may contribute to the mechanism(s) underlying MUD, HAND, and the comorbidity of them. These findings also shade lights on possible pharmacological intervene using K^+^ channel activators and CCR5/CXCR4 inhibitors against Meth and neuroHIV-induced K^+^ channel dysfunction.

## Data Availability

The original contributions presented in the study are included in the article/supplementary material, further inquiries can be directed to the corresponding author.
